# 590 nm LED Irradiation Improved Erythema through Inhibiting Angiogenesis of Human Microvascular Endothelial Cells and Ameliorated Pigmentation in Melasma

**DOI:** 10.3390/cells11243949

**Published:** 2022-12-07

**Authors:** Xiaoxi Dai, Shanglin Jin, Yijie Xuan, Yiwen Yang, Xiaoli Lu, Chen Wang, Li Chen, Leihong Xiang, Chengfeng Zhang

**Affiliations:** Department of Dermatology, Huashan Hospital, Fudan University, 12 Middle Wulumuqi Road, Shanghai 200040, China

**Keywords:** 590 nm LED, photobiomodulation, HMEC-1, angiogenesis, AKT/PI3K/mTOR, melasma

## Abstract

Melasma is a common refractory acquired pigmentary skin disease that mainly affects middle-aged women. The pathogenesis of melasma is still uncertain, while abnormal vascular endothelial cells may play a role. We previously demonstrated the yellow light of light-emitting diodes (LED) could inhibit melanogenesis through the photobiomodulation (PBM) of melanocytes and keratinocytes. In the current study, we investigated the effect of 590 nm LED on the function of human microvascular endothelial cells (HMEC-1). We revealed 0–40 J/cm^2^ 590 nm LED had no toxic effect on HMEC-1 in vitro. 590 nm LED irradiation significantly reduced cell migration, tube formation, as well as the expression of vascular endothelial growth factor (VEGF) and stem cell factor (SCF), a pro-melanogenic factor. Moreover, we illustrated that 590 nm LED inhibited the phosphorylation of the AKT/PI3K/mTOR signaling pathway, and the inhibitory effect on HMEC-1 could be partially reversed by insulin-like growth factor 1 (IGF-1), an AKT/PI3K/mTOR pathway agonist. Besides, we conducted a pilot clinical study and observed a marked improvement on facial erythema and pigmentation in melasma patients after amber LED phototherapy. Taken together, 590 nm LED inhibited HMEC-1 migration, tube formation and the secretion of VEGF and SCF, predominantly through the inhibition of the AKT/PI3K/mTOR pathway, which may serve as a novel therapeutic option for melasma.

## 1. Introduction

Melasma is a common acquired hyperpigmentation skin disease with the clinical manifestation of symmetrical and irregular brown pigmentary macules or patches on the face. The pathogenesis of melasma remains unknown. Previous studies have shown that vascularization might be involved in the development of melasma [1].

Immunohistochemistry evaluation has demonstrated that, compared with perilesional normal skin, the melasma lesion has increased numbers of enlarged blood vessels and higher vascular endothelial growth factor (VEGF) expression, with a positive correlation between the number of vessels and pigmentation [2]. In a total of 100 benign vascular skin lesions, high-magnification digital dermatoscopy revealed a mild to marked hyperpigmentation in 89% cases and marked hyperpigmentation in 22% cases within and surrounding the vascular lesions [3]. At the cellular level, the proliferation of endothelial cells (ECs), which are found in every vascular bed and produce autocrine and paracrine molecules to regulate cell adhesion, as well as vessel permeability, also participate in the modulation of melanogenesis [4]. Regazzetti [3] showed that endothelin 1 (ET-1) released by microvascular endothelial cells increased melanogenesis signaling through the activation of endothelin receptor B and the mitogen-activated protein kinase (MAPK) pathway via extracellular signal-regulated kinase (ERK)1/2 and p38 in melanocytes. Kim [5] showed ECs were activated upon UV exposure to release stem cell factor (SCF), which was elevated in melasma lesions as a melanogenic factor through SCF/c-kit signaling in melanocytes. On the other hand, ECs secreted transforming growth factor-β1 (TGF-β1) and maintained a low level of pigment production under normal physiological conditions [6]. Therefore, both the pathological melanin synthesis in melanocytes and the telangiectasia activity in abnormal ECs should be addressed in the causes of melasma.

Photobiomodulation (PBM), also known as low-level laser therapy (LLLT), mediates nonthermal reactions to regulate biological activities through the absorption of photons in chromophores. Since it was discovered in the 1960s, it has been used to treat a multitude of dermatoses, such as hair regrowth, skin rejuvenation, acne, photoprotection, herpes virus lesions, psoriasis, hypertrophic scars and keloids [7,8]. In terms of the light source for LLLT, it was originally believed that the coherence of the laser was crucial to achieve the therapeutic effect, but recently, this notion has been challenged by the use of light-emitting diodes (LED), which emit non-coherent light over a wider range of wavelengths and have the advantages of being mild, easy to operate and available for home use [7]. For melanin-overproducing skin conditions, Kim [9] showed LED irradiation at 830 nm and 850 nm significantly reduced melanin production and tyrosinase expression via the cyclic AMP (cAMP), AKT and ERK1/2 signaling pathways. In our previous studies, we revealed LED 585 nm yellow light (5, 10, 15, 20 J/cm^2^) did not induce significant changes in cell proliferation and apoptosis, contrary to LED 630 nm red light and LED 420 nm blue light with higher fluence [10]. Furthermore, LED yellow light suppressed melanin content in melanocytes, and the effect was caused by the induction of autophagy [10]. Furthermore, we showed that 585 nm LED could inhibit melanogenesis by upregulating H19 and its derived exosomal miR-675 from keratinocytes [11]. In addition, LED illumination could also modulate the biological function of ECs and angiogenesis, which depended on the wavelength and irradiation energy [12,13]. However, the effect of LED yellow light on the function of ECs remains elusive.

In this research, we demonstrated 590 nm LED inhibited human microvascular endothelial cell (HMEC-1) migration, tube formation and the expression of VEGF and SCF partly via the downregulation of AKT/phosphatidylinositol-3-kinase (PI3K)/mammalian target of the rapamycin (mTOR) pathway. Moreover, we conducted a pilot controlled clinical trial and observed 590 nm LED phototherapy significantly improved facial erythema, as well as pigmentation in melasma patients. Taken together, we illustrated that 590 nm LED alleviated angiogenesis both in vivo and in vitro, through inhibiting HMEC-1 migration, tube formation and the secretion of VEGF via the AKT/PI3K/mTOR pathway, as well as the release of melanogenic factor SCF. Our findings may shed light on melasma treatment by the application of LLLT.

## 2. Materials and Methods

### 2.1. HMEC-1 Acquisition and Cell Culture

HMEC-1 was purchased from Guandao Biological Engineering Co., Ltd. (Shanghai, China). HMEC-1 was inoculated in a T25 culture flask (Corning, Corning, NY, USA) at a density of 5 × 10^5^/mL in a 37 °C, 5 % CO_2_ atmosphere with endothelial cell medium (ScienCell, Carlsbad, CA, USA). The cell line was passaged at a dilution of 1:2–1:3 every 2–3 days.

### 2.2. 590 nm LED Irradiation and Signaling Pathway Agonist Pretreatment

HMEC-1 cells were seeded in 6-well or 96-well plates (Corning), depending on subsequent experiments, for 24 h to ensure cell adhesion. 50 ng/mL insulin-like growth factor 1 (IGF-1) (Peprotech, Rocky Hill, NJ, USA) was added 2 h before 590 nm LED irradiation, if necessary. After washing cells once with Phosphate-buffered solution (PBS) (Biosharp, Shanghai, China), the endothelial cell medium was replaced by Dulbecco’s modified eagle medium (DMEM) without phenol red (Solarbio Science&Technology, Beijing, China). The LED device (590 ± 10 nm, continuous emission mode, 35 mW/cm^2^) used in this study was provided by Xuzhou Kernel Medical Equipment Co., Ltd., Xuzhou, Jiangsu, China. The formula W = P × t was used to compute the irradiation time.

### 2.3. Cell Viability Assay

Cells at a density of 1 × 10^4^ were plated into 96-well plates with three duplicates at each irradiation fluence. 10 μL CCK-8 solution (Biodragon immunotechnologies, Beijing, China) was added 12 h, 24 h and 48 h after 590 nm LED irradiation, and the absorption at 450 nm was measured with a spectrophotometer (Thermo, Waltham, MA, USA). Cell viability was calculated, and replicate experiments were performed using cells of diverse passages.

### 2.4. Flow Cytometry Analysis

HMEC-1 cells were grown in 6-well plates at a density of 5 × 10^5^ and irradiated by different dosages of 590 nm LED. After 24 h, cells were isolated with 0.25% trypsin without EDTA (Gibco, Grand Island, NY, USA), centrifuged, resuspended with 4 °C PBS. Subsequently, irradiated cells were washed, stained with Annexin-V FITC & PI (BD Biosciences; San Jose, CA, USA), measured with a C6 flow cytometer (BD) and analyzed using FlowJo (BD). For reactive oxygen species (ROS) measurement, gathered cells were resuspended in serum-free medium containing DCFH-DA (1:1000, Biodragon immunotechnologies), kept at 37 °C for 20 min, washed and detected by flow cytometry.

### 2.5. Wound Healing Assay

The scratch wound assay has been the most common method to measure cell migratory capacity in vitro. Cells at a density of 5 × 10^5^ in the logarithmic growth phase were seeded in 6-well plates with three equidistant horizontal lines on the bottom and incubated to 100% confluence. Three equispaced vertical lines were scratched perpendicular to the marked lines at the bottom with 200 μL pipette tips (Axygen, Tewksbury, MA, USA), then exfoliated cells were rinsed with PBS. After 590 nm LED irradiation, cells were cultured in serum-free medium and were photographed at the same position using an inverted microscope (Nikon, Tokyo, Japan) at 0 h, 24 h, and 48 h. The scratch area and cell migration rate were calculated using Image J (NIH, Bethesda, MD, USA).

### 2.6. Tube Formation Assay

A pre-chilled 96-well plate was coated with Matrigel matrix (Corning) and incubated at 37 °C for 30 min to allow the Matrigel solution to solidify. After LED irradiation, the cells were collected and plated into the pre-coated 96-well plate at a density of 5 × 10^4^. Eight hours later, brightfield photos were taken under a 200× inverted microscope, and the number of meshes were measured by Image J. Three independent experiments were performed for each fluence.

### 2.7. Real-Time Quantitative Polymerase Chain Reaction (RT-qPCR)

For total RNA isolation, Trizol (Invitrogen, Carlsbad, CA, USA), chloroform (China sinopharm, Shanghai, China) and isopropanol (Sangon biotech, Shanghai, China) were added into irradiated cells, subsequently followed by 75% ethanol (China sinopharm) rinsing. The reverse transcription reaction system was performed according to the manufacturer’s protocol of the PrimeScript RT Master Mix kit (Takara, Tokyo, Japan). The synthesized cDNA samples were amplified and detected in the RT-qPCR system under the instruction of TB Green Premix Ex Taq II kit (Takara) in QuantStudio 6 (Thermo) (Tables S1–S5, RT-qPCR protocol).

### 2.8. Enzyme-Linked Immunosorbent Assay (ELISA)

According to the manufacturer’s instructions of the human VEGF and SCF ELISA kit from Multisciences Biotech, Hangzhou, Zhejiang, China, standard vials were dissolved and doubly diluted for the standard curve. The obtained supernatant of irradiated cells was centrifuged, added into plates, and incubated with antigens, horseradish peroxidase (HRP)-avidin and TMB substrate. The absorption at wavelengths of 450 nm and 630 nm was measured, and the concentration of the samples was calculated according to the standard curve and dilution multiple.

### 2.9. Western Blot

Irradiated cells in 6-well plates were lysed on ice using RIPA mixed with 1% PMSF (Beyotime, Shanghai, China). Loading buffer was added into the supernatant after centrifugation, and the extract was heated to 99 °C to be denatured. The obtained protein samples were separated in the SDS-PAGE system (Genscript, Nanjing, Jiangsu, China), transferred to polyvinylidene difluoride (PVDF) membranes (Millipore, Boston, MA, USA), and incubated with antibodies against AKT, Phospho-AKT, PI3K, mTOR, β-actin (Cell Signaling Technology, Boston, MA, USA, 1:1000–1:2000), AKT (Cell Signaling Technology, 1:1000; Abcam, Cambridge, UK, 1:10,000), Phospho-PI3K (Affinity Biosciences, Liyang, Jiangsu, China, 1:1000), and HRP-labeled goat anti rabbit IgG (Beyotime, 1:5000). The protein expression was detected using enhanced chemiluminescence reagent (Biosharp) in an image analyzer (Tanon, Shanghai, China).

### 2.10. Comet Assay

The condition of deoxyribonucleic acid (DNA) damage after irradiation was detected using the comet assay kit (Nanjing Jiancheng Bioengineering Institute, Nanjing, Jiangsu, China). HMEC-1 in 6-well plates was irradiated, isolated, centrifugated, and resuspended in PBS. Agarose slides were prepared by the first layer of 100 uL 0.5% normal-melting agarose (NMA), the second layer of 75 uL 0.7% low-melting agarose (LMA) mixing with 10 μL cell suspension and the third layer of 75 uL LMA. After cells lysis, DNA was unwound in alkaline buffer and determined by electrophoresis, neutralization and PI staining. Fluorescence photographs taken were analyzed by Image J. DNA damage was classified into five grades based on tailDNA percent according to manufacturer’s instruction.

### 2.11. 590 nm LED Phototherapy

The clinical research was approved by the Ethics Committee of Huashan Hospital, Fudan University (protocol code: KY2019-515), and informed consent was obtained from all participants. A total of ten patients with mild to severe melasma were treated with the amber light LED device (product code: KN-7000D, wavelength: 585 ± 10 nm, power density: 20 mW/cm^2^, irradiation area: 850 cm^2^ ± 10%, donated by Xuzhou Kernel Medical Equipment Co.). Each patient was irradiated with 20 J/cm^2^ LED, approximately 1000 s, once a week for eight consecutive sessions. Follow-up was performed every four weeks during the course, one month and three months after the end of treatment. Images were taken using the VISIA Complexion Analysis System (Canfield Scientific Co., Parsippany, NJ, USA); the melanin index (MI) and erythema index (EI) were recorded using the Mexameter dermaspectrophotometer (Cortex Technology, Hadsaund, Denmark) as well. The melasma area severity index (MASI) [14] was conducted by two dermatologists independently, according to the formula. All reported adverse effects were recorded.

### 2.12. Statistical Analysis

GraphPad Prism 6 (GraphPad Software, La Jolla, CA, USA) or SPSS Statistics 26.0 (IBM, Armonk, NY, USA) were used to perform statistical analysis on collected data. One-way ANOVA or paired *t*-tests were used for the statistical analysis in appropriate quantitative data. Otherwise, the Wilcoxon test was conducted for the nonparametric test. *p* < 0.05 was considered statistically significant.

## 3. Results

### 3.1. Effects of 590 nm LED on Cell Viability of HMEC-1

**Figure 1:**
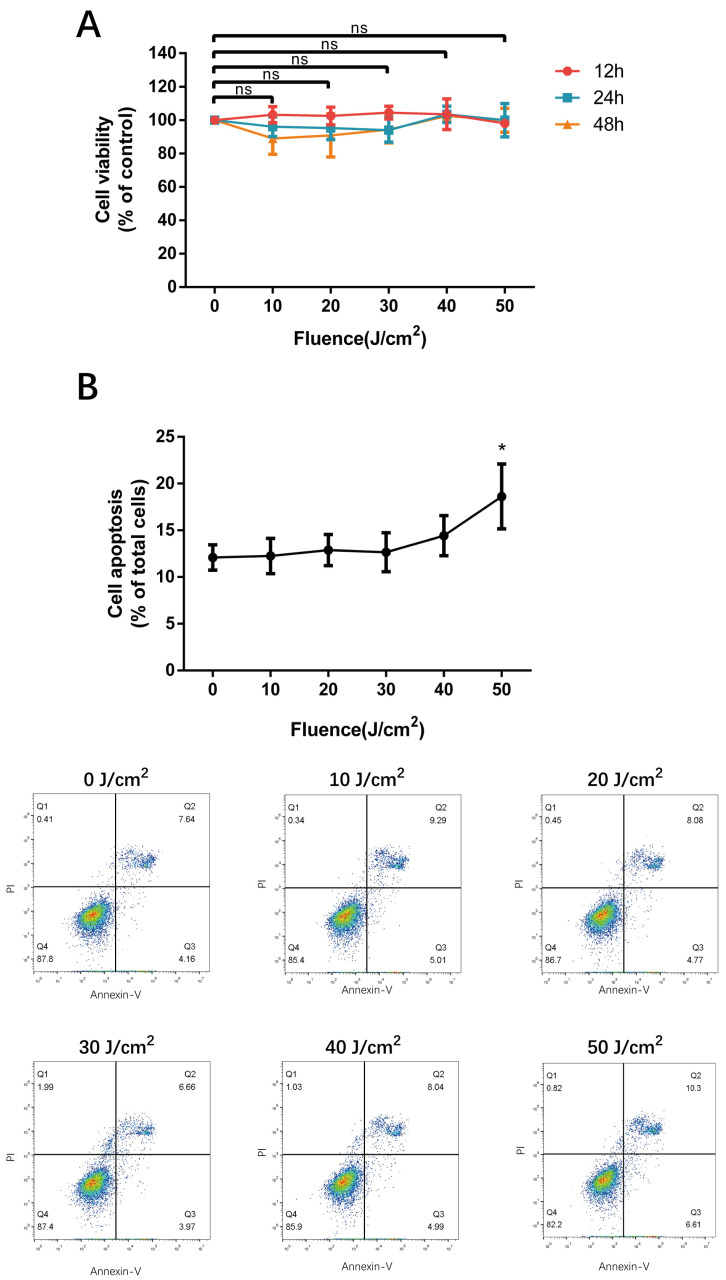
The effect of 590 nm LED irradiation on cell viability and cell apoptosis of HMEC-1. (**A**) Cell viability of HMEC-1 measured using CCK-8 assay 12 h, 24 h and 48 h after 0–50 J/cm^2^ 590 nm LED illumination. Results were normalized to the control. (**B**) Apoptosis of irradiated cells detected by flow cytometry (24 h, Annexin-V FITC & PI staining). Data are presented as means ± SD in independent experiments. *n* = 3; *, *p* < 0.05. n.s., no significance.

To explore the influence of 590 nm LED on the cell viability of HMEC-1, a CCK-8 assay was performed 12 h, 24 h and 48 h after 0–50 J/cm^2^ LED irradiation. The results show that cell viability did not change significantly after 0–50 J/cm² LED irradiation (*p* > 0.05), as shown in Figure 1A. In terms of cell apoptosis, flow cytometry with Annexin-V FITC & PI staining was conducted 24 h after LED irradiation and revealed no statistical difference among fluences of 0 to 40 J/cm², whereas 50 J/cm² LED irradiation increased the cell apoptosis rate by 53.9 % (*p* = 0.035), as shown in Figure 1B. Therefore, we determined to evaluate cell function with the irradiation fluences of 0–40 J/cm^2^ in subsequent experiments.

### 3.2. 590 nm LED Inhibited Migration of HMEC-1

**Figure 2:**
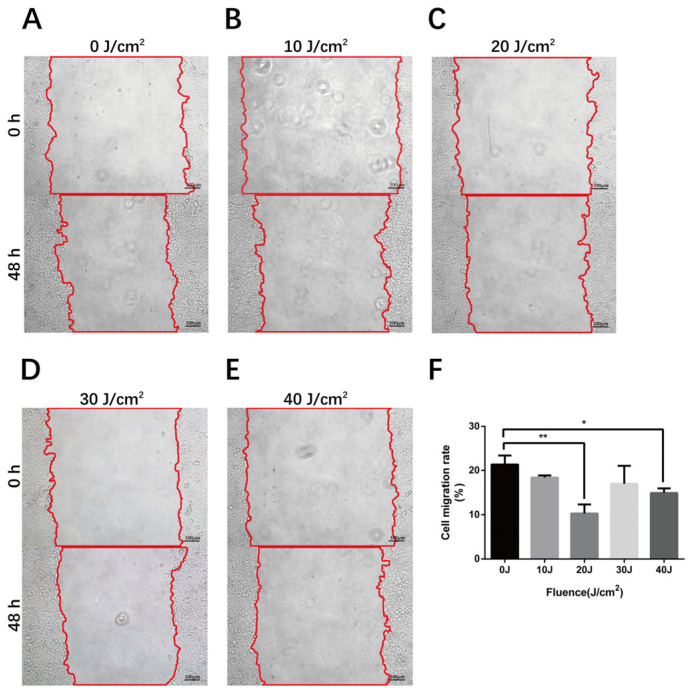
The influence of 590 nm LED on cell migration of HMEC-1. (**A**–**E**) The wound healing area 48 h after 0–40 J/cm^2^ LED irradiation. (**F**) Cell migration rate 48 h after 0–40 J/cm^2^ LED irradiation. Results from three independent experiments are presented as means ± SD. *, *p* < 0.05; **, *p* < 0.01. Bar, 100 μm.

The process of angiogenesis depends on the proliferation and migration of ECs. To learn more about the influence of 590 nm LED on the cell migration of HMEC-1, we applied wound healing assay to detect cell mobility 48 h after 0–40 J/cm^2^ LED irradiation (Figure 2A–E). The migration rate of cells which were irradiated by 20 J/cm^2^ LED decreased from 21.3 % ± 2.041 to 10.3 % ± 2.039 (*p* = 0.005). Moreover, the cell migration rate reduced to 15.0 % ± 1.045 (*p* = 0.044) after 40 J/cm^2^ LED exposure, as shown in Figure 2F.

### 3.3. 590 nm LED Suppressed Tube Formation of HMEC-1

**Figure 3:**
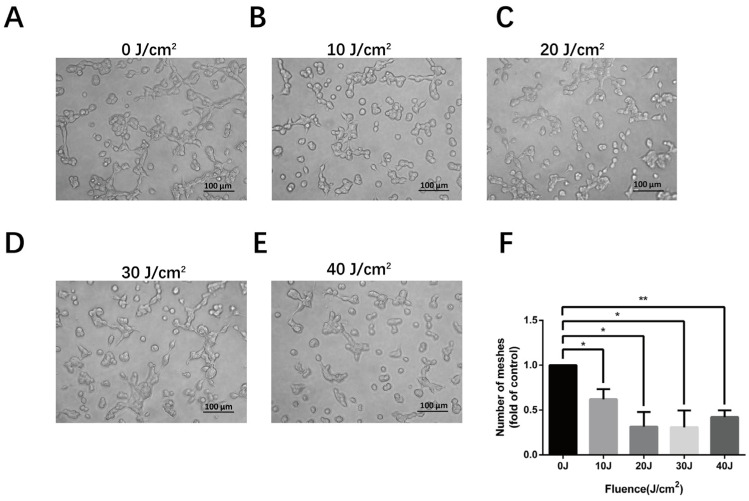
The suppression of tube formation in HMEC-1 after 590 nm LED irradiation. (**A**–**E**) The cell tube formation condition 8 h after 0–40 J/cm^2^ LED irradiation. (**F**) The comparison of mesh numbers. Data are normalized to the control and are shown as mean values with SD from three independent tests. *, *p* < 0.05; **, *p* < 0.01. Bar, 100 μm.

An EC tube formation assay could be used to measure angiogenesis in vitro in a fast, reproducible and quantifiable manner [15]. To observe the tube-forming ability of HMEC-1 irradiated by 590 nm LED, we performed a tube formation experiment to simulate capillary angiogenesis. Our results show that the number of meshes declined markedly after irradiation (0.621 ± 0.112-fold of control, *p* = 0.028 at 10 J/cm^2^; 0.314 ± 0.164-fold of control, *p* = 0.019 at 20 J/cm^2^; 0.308 ± 0.188-fold of control, *p* = 0.024 at 30 J/cm^2^; 0.422 ± 0.075-fold of control, *p* = 0.006 at 40 J/cm^2^), indicating PBM inhibited the tube formation of ECs in vitro, especially at the fluences of 20–40 J/cm^2^ (Figure 3).

### 3.4. 590 nm LED Reduced Release of VEGF and SCF

**Figure 4:**
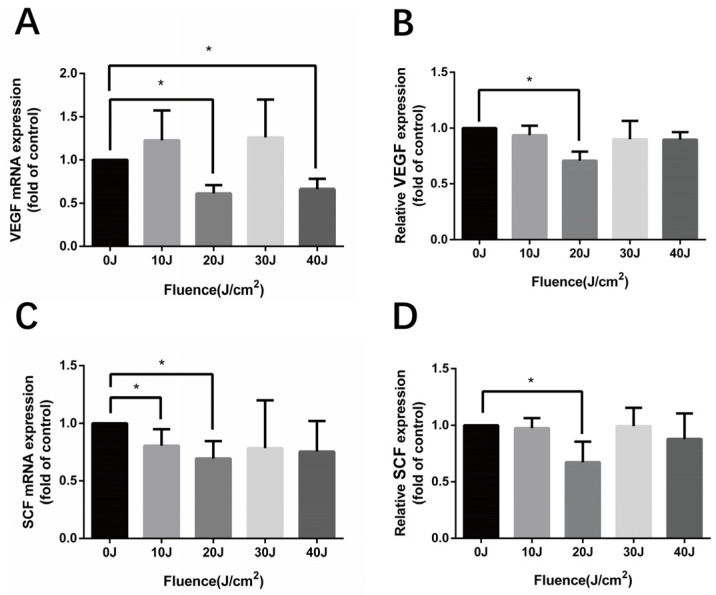
The expression of VEGF and SCF in HMEC-1 after 590 nm LED irradiation. (**A**,**B**) The synthesis and release of VEGF in HMEC-1 detected by RT-qPCR and ELISA after 590 nm LED irradiation (0–40 J/cm^2^, 24 h). (**C**,**D**) SCF synthesis and secretion of irradiated HMEC-1 48 h later. Data of treatment groups are normalized to the control and independent experiment results are expressed as means ± SD. (**A**,**B**) *n* = 3; (**C**,**D**) *n* = 5. *, *p* < 0.05. VEGF, vascular endothelial growth factor; SCF, stem cell factor.

Altered angiogenesis and melanogenesis are frequently found in melasma patients. We thus explored the expression of angiogenesis-related and melanogenesis-related factors after 590 nm LED treatment. Indeed, LED irradiation at a 590 nm wavelength reduced the levels of VEGF and SCF in HMEC-1. As shown in Figure 4B,D, the inhibitory impact was not completely dose-dependent but was the most obvious at the dose of 20 J/cm^2^ (VEGF 0.708 ± 0.081-fold of control, *p* = 0.025; SCF 0.673 ± 0.182-fold of control, *p* = 0.016). By contrast, the mRNA and protein expression of ET-1, as well as TGF-β1, remained unchanged after LED irradiation (*p* > 0.05) (Figure S1).

### 3.5. 590 nm LED Inhibited Angiogenesis Predominantly via AKT/PI3K/mTOR Pathway

**Figure 5:**
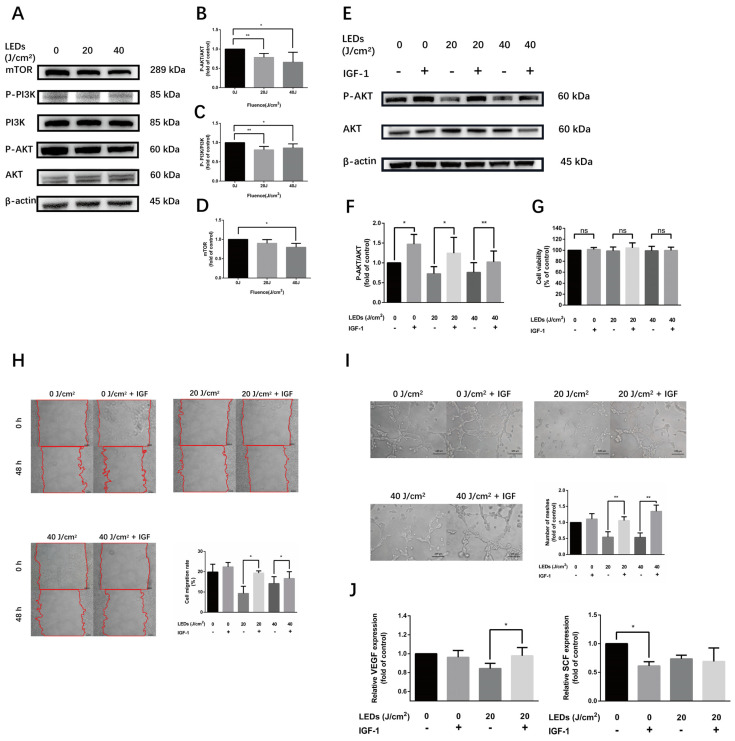
590 nm LED inhibited angiogenesis of HMEC-1 predominantly via AKT/PI3K/mTOR pathway. (**A**–**D**) The phosphorylation level of AKT/PI3K/mTOR pathway after LED illumination (20, 40 J/cm^2^, 0.5 h). (**E**,**F**) The phosphorylation level of AKT after LED irradiation (20, 40 J/cm^2^) alone or pretreated with IGF-1 (50 ng/mL, 2 h). (**G**) Cell viability of irradiated cells or with IGF-1 pretreatment. (**H**) Cell migration (48 h) of wound healing assay after LED illumination alone or pretreated with IGF-1. Bar, 100 μm. (**I**) Number of meshes (8 h) of HMEC-1 after LED irradiation alone or with IGF-1 pretreatment. Bar, 100 μm. (**J**) The secretion of VEGF and SCF from cells irradiated by 20 J/cm^2^ LED alone or with IGF-1 pretreatment detected by ELISA. (**B**–**D**,**F**,**G**,**I**,**J**) Results of irradiated groups are normalized to the control. Data are shown as means ± SD. (**A**–**F**) n = 5; (**G**–**J**) *n* = 3. *, *p* < 0.05; **, *p* < 0.01. LED, light-emitting diodes; IGF-1, insulin-like growth factor 1; VEGF, vascular endothelial growth factor; SCF, stem cell factor; n.s., no significance.

To illustrate the underlying mechanism of 590 nm LED on the biological function of HMEC-1, the phosphorylation level of the AKT/PI3K/mTOR pathway was measured after irradiation. The results show that 590 nm LED significantly inhibited the AKT/PI3K/mTOR pathway, which could be reversed by a 50 ng/mL AKT pathway agonist IGF-1 pretreatment without an effect on cell activity (Figure 5A–G). Furthermore, IGF-1 could attenuate the inhibitory effect of 590 nm LED on the cell migration and tube formation of HMEC-1 (Figure 5H,I). The suppression of 20 J/cm^2^ LED on the release of VEGF from HMEC-1 cells was also reversed by IGF-1 pretreatment. Conversely, IGF-1 addition diminished the expression of SCF secretion, suggesting that the inhibition effect of 590 nm LED was unrelated to the downregulation of the AKT/PI3K/mTOR pathway (Figure 5J). Taken together, 590 nm LED inhibited the angiogenesis of HMEC-1 predominantly through the AKT/PI3K/mTOR pathway. 

Studies revealed that AKT could be destroyed by the ubiquitin-mediated protein degradation pathway, and cellular ROS could increase Mitochondrial E3 ubiquitin protein ligase 1 (MUL1) expression, a negative regulator of AKT ubiquitination [16]. Therefore, we detected the ROS level in irradiated cells to explore the potential mechanism of AKT pathway downregulation, and the results show no statistical difference between the control group and illuminated groups, as shown in Figure S2A,B. 

To monitor the safety of LED treatment, DNA damage in HMEC-1 was evaluated by comet assay. No obvious comet phenomenon was observed, and the tailDNA percent remained unchanged after LED exposure (Figure S2C,D). 

### 3.6. 590 nm LED Ameliorated Pigmentation and Facial Erythema in Melasma

**Figure 6:**
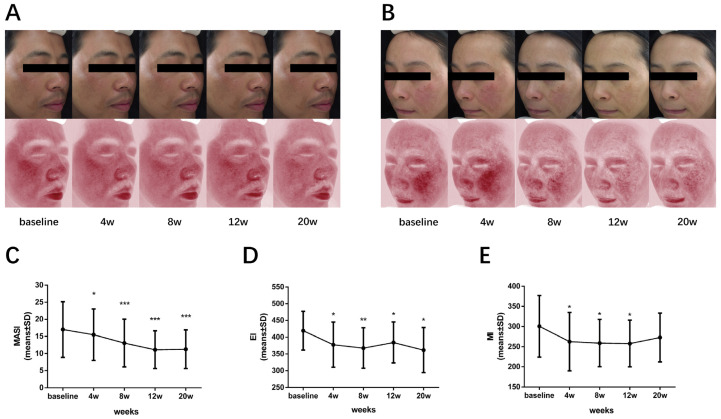
Improvement of hyperpigmentation and erythema in melasma patients after 590 nm LED light therapy. (**A**,**B**) Representative photographs (upper panel) and VISIA images (lower panel) of two melasma patients, showing significant improvement of hyperpigmentation and erythema after phototherapy. The change in mean MASI score (**C**), EI (**D**) and MI (**E**) of all patients enrolled during the course, shown as means ± SD. *n* = 10. *, *p* < 0.05; **, *p* < 0.01; ***, *p* < 0.001. MASI, melasma area severity index; EI, erythema index; MI, melanin index.

In order to explore the potential therapeutic effect of 590 nm LED on melasma, we further conducted a single-center pilot clinical observation. A total of ten patients diagnosed with mild to severe melasma were enrolled and treated with 590 nm LED phototherapy with a 20 J/cm^2^ dosage, once a week, for eight weeks consecutively. The subject characteristics are shown in Table S6. Clinical images indicated a visible improvement in both facial erythema and hyperpigmentation (Figure 6A,B). When it comes to objective assessment, the mean MASI score significantly decreased from 17.020 ± 8.140 to 13.050 ± 6.963 in week eight (*p* < 0.001), with a 23.3 % improvement, as shown in Figure 6C. Meanwhile, the EI and MI of these patients were significantly decreased compared with the baseline in week eight (EI 419.500 ± 57.770 to 367.700 ± 60.470, *p* = 0.003; MI 300.500 ± 76.400 to 258.900 ± 58.720, *p* = 0.035; Figure 6D,E). To be noted, no participants reported worsened symptoms or a severe adverse reaction during the whole treatment. Collectively, these results tentatively verify the efficacy and safety of 590 nm LED phototherapy to ameliorate the hyperpigmentation and facial erythema in melasma patients.

## 4. Discussion

**Figure 7:**
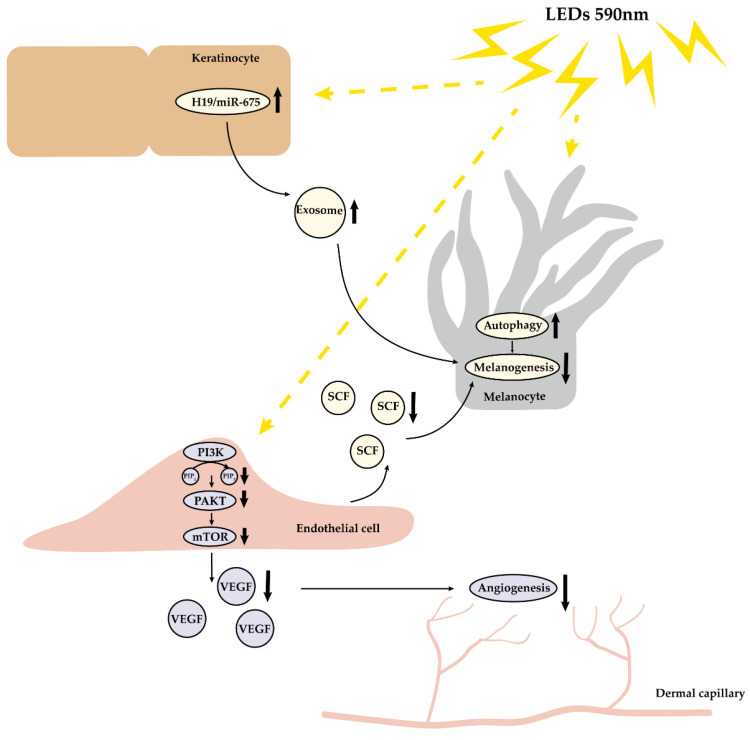
Multiple effects of 590 nm LED photobiomodulation on the microenvironment of skin. 590 nm LED alleviated angiogenesis through inhibiting cell migration, tube formation and VEGF release in endothelial cells, predominantly via the downregulation of the AKT/PI3K/mTOR pathway. In addition, LED suppressed melanogenesis via inducing autophagy in melanocytes, upregulating the H19/miR-675 axis and its exosome in keratinocytes, probably through decreasing the secretion of SCF in endothelial cells. LED, light-emitting diodes; VEGF, vascular endothelial growth factor; SCF, stem cell factor.

Light-emitting diode treatment is an emerging non-thermal light therapy modality. We previously uncovered that yellow-light LED decreased melanin synthesis through the direct regulation of melanocytes, as well as the indirect effect on keratinocytes [10,11]. Although it was clinically observed that LED phototherapy might improve the erythema and pigmentation of melasma, clinical trials and underlying mechanism research are still missing. To this end, we initiated this study and revealed that 590 nm LED inhibited cell migration, tube formation, as well as the synthesis and secretion of VEGF and SCF in HMEC-1, partially via downregulating the AKT/PI3K/mTOR signaling pathway. Therefore, we concluded that, besides the effects of PBM on melanocytes and keratinocytes, 590 nm LED inhibited angiogenesis through the suppression of microvascular endothelial cells via the AKT/PI3K/mTOR pathway and reduced the release of SCF, which might serve as a new strategy for treating melasma from three aspects (Figure 7).

The primary chromophore of PBM is the electron transport chain located in the mitochondrial membrane; in particular, the enzyme cytochrome c oxidase (CCO), opsin 3, flavins, flavoproteins and porphyrins also play a role [17,18,19]. The number of mitochondria in cells and tissues varies widely to correlate with the metabolic requirement, and cells with higher numbers of mitochondria respond better to PBM than cells with lower numbers of mitochondria [20]. Studies on various wavelengths and different therapeutic dosages of PBM’s effects on fibroblasts and skin tissue, possessing fewer mitochondria, have been reported, whereas the effect of 590 nm LED on vascular endothelial cells remains unclear.

Generally, a longer wavelength penetrates the dermis to a greater extent than shorter wavelengths [21]. The Roscoe–Bunsen law of reciprocity expounds that the most important parameter of PBM, the power density (irradiance) measured in mW/cm^2^ and the energy density (fluence) measured in J/cm^2^, is the total quantity of photons absorbed by the target cells, which is a fundamental concept of LLLT [20,22]. In this study, we used a 590 nm LED irradiation equipment with 35 mW/cm^2^ irradiance for in vitro experiment and a yellow LED device with a power density of 20 mW/cm^2^ for clinical observation. In regard to the energy density used in the clinical trial, we chose the dose of 20 J/cm^2^ according to the non-toxic irradiation dose in a prior keratinocyte experiment [11]. The effect of mode of light delivery in PBM remains controversial [23], but the continuous wave mode was used in our study due to the function of the experimental irradiator, and we will explore further the differences between continuous and pulsed emission modes.

When it comes to the LED modulation of HMEC-1, the Arndt–Schultz law, proposed near the end of the 19th century, states in its original form that “For every substance, small doses stimulate, moderate doses inhibit, and large doses kill”, which has been used as another convenient concept to explain the cellular and tissue interactions with light [20]. Indeed, cell viability detected by the CCK-8 assay was not altered significantly after 0–50 J/cm² LED irradiation, which is consistent with the safe and mild efficacy of 590 nm LED phototherapy observed in clinical practice. However, flow cytometry revealed the cell apoptosis rate increased under the fluence of 50 J/cm², which might indicate that HMEC-1 merely underwent early apoptosis with an undamaged cell membrane. Perhaps it could be due to the different sensitivities of detection techniques. Additionally, the magnitude of PBM depends on the physiological state of the cell at the moment of irradiation, and there exists undetectable effects, as well as the variability of the results reported in the literature [24], which might explain why the inhibitory effect of 590 nm LED on cell phenotype and function, such as cell migration, tube formation and secretion, was not absolutely dose-dependent. Therefore, our findings actually accord with the basic mechanism and characteristics of PBM.

Furthermore, we found that the autocrine potent angiogenic molecule VEGF was reduced notably by 590 nm LED. It has been revealed that VEGF stimulates EC prostacyclin production, which is the direct precursor of prostaglandin E2, an activator of melanocyte derived from keratinocyte [25,26]. In addition, normal human melanocytes constitutively express functional VEGF receptor (VEGFR)-1, VEGFR-2, and neuropilin-1, among which VEGFR-2 expression is induced by ultraviolet irradiation [27]. Therefore, VEGF might potentially participate in the melanogenesis regulation of melanocytes. Moreover, 590 nm LED decreased the secretion of SCF, a paracrine factor from keratinocytes and fibroblasts which induces specific internal signaling pathways of melanogenesis in melanocytes, including the cAMP/protein kinase A (PKA), MAPK, Wnt/β-catenin, AKT/PI3K and SCF/c-Kit signaling pathways [28]. Here, we demonstrated that ECs were another source of SCF in the dermis, which is in line with previous research [5]. 

Angiogenesis, the process of new blood vessel formation from existing ones, depends on the proliferation and migration of vascular endothelial cells under the regulation of multiple factors. The activation of the AKT/PI3K/mTOR pathway in tumor cells has been found to increase VEGF secretion and plays an essential role in angiogenesis regulation by modulating endothelial cell migration, the formation of structurally abnormal blood vessels, as well as the expression of nitric oxide and angiopoietins in normal tissues and in cancers [29]. Previous studies found that PBM on vascular endothelial cells was related to the regulation of the AKT/PI3K signaling pathway [12]. The classic AKT agonist, IGF-1, binds to the IGF-1 receptor and induces AKT/PI3K pathway phosphorylation [30]. In the current study, we proved 590 nm LED downregulated the phosphorylated level of the AKT/PI3K/mTOR pathway in HMEC-1. Moreover, the inhibitory effect of 590 nm LED on HMEC-1 could be reversed by IGF-1, indicating that such an inhibitory effect was achieved by suppressing the AKT/PI3K/mTOR pathway. 

LLLT has been widely used in clinical practice. It has been reported that PBM could alleviate skin pigmentation and erythema. During the treatment of facial acne with LED devices, alternating blue (415 nm) and red (633 nm) light, Lee [31] found the melanin level decreased significantly after the red light irradiation in contrast with blue light, whereas both wavelengths of light produced an overall statistically significant decrease in the melanin level. In the process of skin rejuvenation with 590 nm LED PBM in over 300 patients, Weiss [32] observed a softening of the skin texture and a reduction in roughness and fine lines in 90% of patients, as well as a global improvement of facial texture, fine lines, background erythema and pigmentation noted in physician treatment records in 60% of patients. In our study, we observed a significant improvement of erythema, pigmentation and skin texture in melasma patients after 590 nm LED treatment.

Our study had several limitations. Firstly, only a single-cell cytological model of HMEC-1 was performed, which was incapable of imitating adequately vascular and pigmentary regulation in human skin. A UV-induced 3D co-culture model or an artificial skin model may be required to further investigate the interaction of ECs and melanocytes in melasma. Secondly, we could not determine the potential mechanism of LLLT on SCF suppression, which could be an interesting research direction. Lastly, the sample size of our pilot clinical observation was small so that it could not allow us to perform a sub-group analysis. More prospective, randomized controlled clinic trials with larger sample sizes are needed to further evaluate the efficacy and safety of 590 nm LED light therapy in treating melasma.

The pathogenesis of melasma is complicated and the main drawback of current therapy strategy is the indeterminable efficacy and prolonged course with a high recurrence rate. Despite the forementioned limitations, considering its safe, continuable and portable character, 590nm LED phototherapy may be ideal for the treatment and maintenance control of melasma, especially for those with erythema and telangiectasis.

## 5. Conclusions

In conclusion, LED with a wavelength of 590 nm alleviated angiogenesis through inhibiting HMEC-1 migration, tube formation, as well as the synthesis and secretion of VEGF through the AKT/PI3K/mTOR pathway and might suppress melanogenesis via decreasing the release of SCF, which could be a novel therapeutic modality for melasma.

## Data Availability

The datasets supporting the results in this research are available from the corresponding author upon reasonable request.

## Supplementary Materials

The following supporting information can be downloaded at: https://www.mdpi.com/article/10.3390/cells11243949/s1, Tables S1–S5. RT-qPCR protocol; Table S6. Subject characteristics; Figure S1. The mRNA and protein expression of ET-1 and TGFβ1 in HMEC-1 24 h after 590 nm LED irradiation; Figure S2. The reactive oxygen species (ROS) level and DNA damage condition in HMEC-1 24 h after 590 nm LED irradiation.
