# Supplementary material for: 590 nm LED Irradiation Improved Erythema through Inhibiting Angiogenesis of Human Microvascular Endothelial Cells and Ameliorated Pigmentation in Melasma
Source: Cells. 2022 Dec 7;11(24):3949. doi: 10.3390/cells11243949 (PMC9776419; doi:10.3390/cells11243949)
Supplement: Supplementary file 1 [file cells-11-03949-s001.zip › cells-2078827-supplementary.pdf]

Table S1. Primer list

Gene	Forward Primer	Reverse Primer
VEGF	5′CAG AAG GAG GAG GGC AGA A 3′	5′GTC TCG ATT GGA TGG CAG TAG 3′
SCF	5′CTG TTC AAA GCA AAT CCA GAT 3′	5′ AGG AAC CAC AGC ATG TTA GAG 3′
TGFβ1	5′ACC TGA ACC CGT GTT GCT CT 3′	5′CGC CAG GAA TTG TTG CTG TA 3′
ET-1	5′GAG AAA CAG CGT CAA ATC ATC 3′	5′ CCG AAG GTC TGT CAC CAA T 3′

Primers all from BioTNT, Shanghai, China. VEGF, vascular endothelial growth factor; SCF, stem cell factor; ET-1, endothelin 1; TGFβ1, transforming growth factor-β1.

Table S2. 10 μL reserve transcription system

Reagent	Dosage
5 × PrimeScript RT Master Mix	2 μL
Total RNA	500 ng
RNase Free dH_2O	up to 10 μL

Table S3. Reverse transcription protocol

Step	Condition
Reverse transcription reaction	37 °C, 15 min
Inactivation	85 °C, 5 s
4 °C hold	

Table S4. 10 μL RT-qPCR system

Reagent	Dosage
2 × TB Green Premix Ex Taq II	5 μL
PCR Forward Primer	0.4 μL
PCR Reverse Primer	0.4 μL
ROX Reference Dye or Dye II	0.2 μL
cDNA	1 μL
RNase Free dH_2O	3 μL

Table S5. Two step RT-qPCR protocol

Step	Condition
Initial denaturation	Reps: 1; 95 °C, 30 s
PCR reaction	Reps: 40; 95 °C, 5 s; 60 °C, 30–34 s

Reps, Repetitions.

Table S6. Subject characteristics

Characteristic	Total (*n* = 10)
Females, *n* (%)	9 (90.00)
Age, mean (SD), y	43.80 (5.29)
Fitzpatrick skin type, *n* (%)	
III	4 (40.00)
IV	6 (60.00)
Duration of melasma (mean, SD), y	4.80 (1.23)

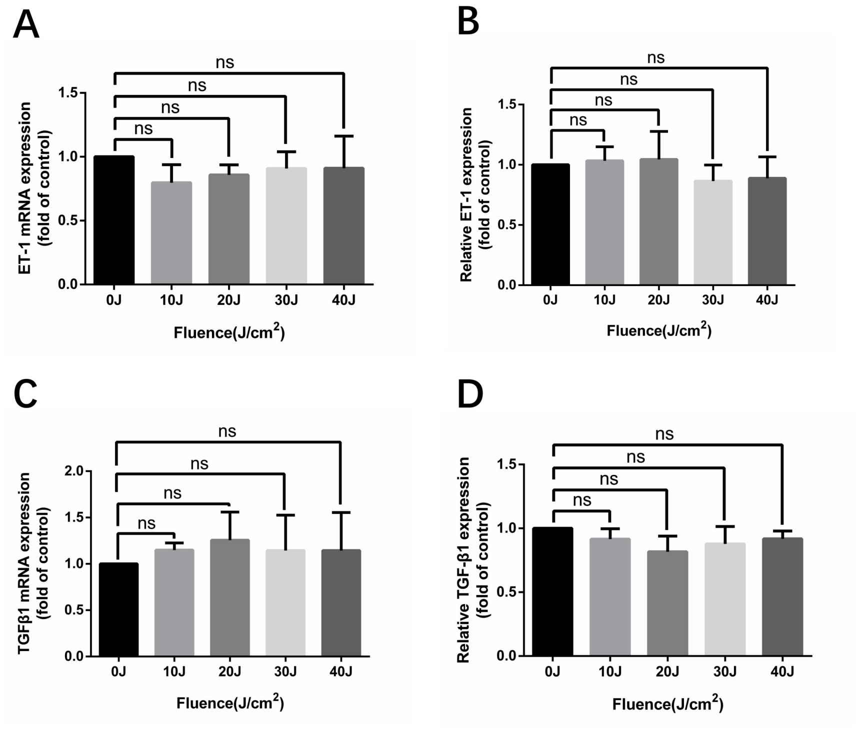

Figure S1. The mRNA and protein expression of ET-1 and TGFβ1 in HMEC-1 24 h after 590 nm LED irradiation. (**A**,**B**) The synthesis and release of ET-1 in HMEC-1 detected by RT-qPCR and ELISA. (**C**,**D**) The synthesis and release of TGFβ1 in HMEC-1. Data of irradiated groups are normalized to the control and three independent experiment results are expressed as means ± SD. ET-1, endothelin 1; TGFβ1, transforming growth factor-β1; n.s., no significance.

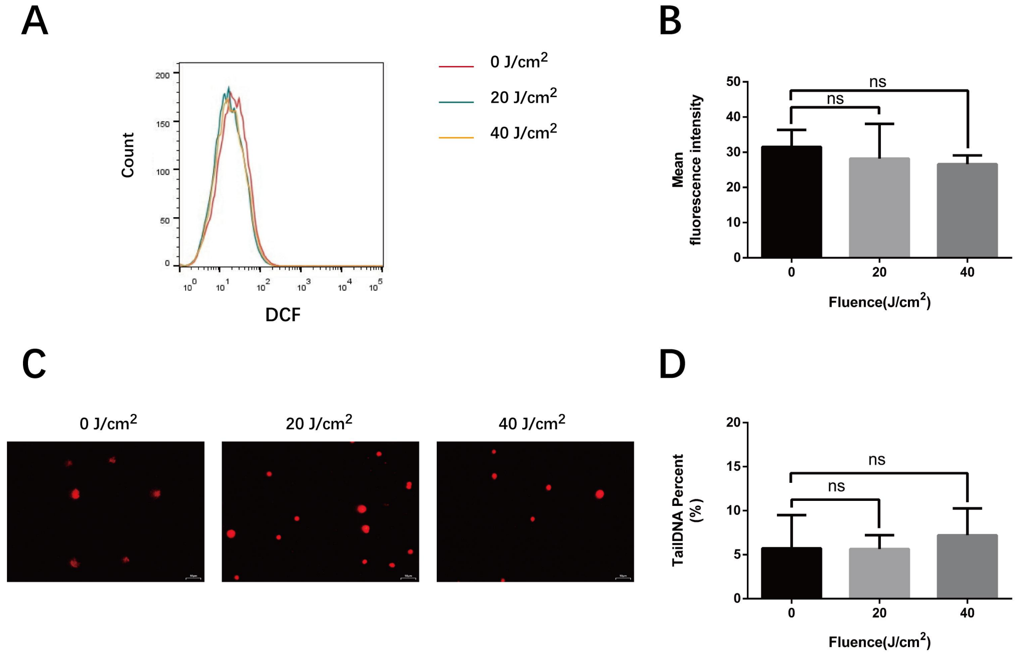

Figure S2. The reactive oxygen species (ROS) level and DNA damage condition in HMEC-1 24 h after 590 nm LED irradiation. (A,B) ROS-positive cells and mean fluorescence intensity measured by flow cytometry analysis (dyed with DCF-DA). (C,D) Fluorescence photographs and tailDNA percent of irradiated cells in comet assay to measure DNA damage. B-D Three independent experiments were performed and results are shown as means ± SD.
